# Adults with patellofemoral pain do not exhibit manifestations of peripheral and central sensitization when compared to healthy pain-free age and sex matched controls – An assessor blinded cross-sectional study

**DOI:** 10.1371/journal.pone.0188930

**Published:** 2017-12-08

**Authors:** Michael Skovdal Rathleff, Camilla Rams Rathleff, Aoife Stephenson, Rebecca Mellor, Mark Matthews, Kay Crossley, Bill Vicenzino

**Affiliations:** 1 Research Unit for General Practice in Aalborg, Department of Clinical Medicine at Aalborg University, Aalborg University, Aalborg, Denmark; 2 Orthopaedic Surgery Research Unit, Aalborg University Hospital, Aalborg, Denmark; 3 University of Queensland, School of Health and Rehabilitation Sciences: Physiotherapy, Brisbane, Australia; 4 College of Science, Health and Engineering. La Trobe University, Bundoora, Australia; Stanford University School of Medicine, UNITED STATES

## Abstract

Patellofemoral Pain (PFP) is highly prevalent among adults and adolescents. Localized mechanical hyperalgesia around the knee and tibialis anterior have been observed in people with PFP, but limited knowledge of potential manifestations of central sensitisation exists. The aims of this study were to study conditioned pain modulation (CPM) and wide-spread hyperalgesia in adults with PFP. This assessor-blinded cross-sectional study design compared CPM and mechanical pressure pain thresholds (PPT) between 33 adults (23 females) diagnosed with PFP and 32 age and sex matched pain-free controls. The investigator taking the PPT measurements was blinded to which participants had PFP. PPTs were reliably measured using a Somedic hand-held pressure algometer at three sites: 1) The centre of the patella, 2) the tibialis anterior muscle and 3) a remote site on the lateral epicondyle. For the assessment of CPM, experimental pain was induced to the contralateral hand by immersion into a cold water bath (conditioning stimulus), and assessment of PPTs (the test stimulus) was performed before and immediately after the conditioning stimulation. On average, the CPM paradigm induced a significant increase in PPTs across the three sites (6.3–13.5%, P<0.05), however there was no difference in CPM between young adults with PFP compared to the control group, (*F*(1,189) = 0.39, P = 0.89). There was no difference in mechanical PPTs between the two groups (*F*(1,189) = 0.03, P = 0.86). Contrary to our *a-priori* hypothesis, we found no difference in CPM or PPT between young adults with PFP and age and sex matched pain-free controls.

## Introduction

One of the most long-lasting and troublesome knee conditions among adolescents and adults is patellofemoral pain (PFP). Patellofemoral pain is a clinical diagnosis of pain felt anteriorly around the patella, which increases during prolonged sitting, squatting, kneeling and stair climbing[1]. The prevalence of PFP among adolescents and young adults is 7% and 9% respectively [2–4] and may account for up to 17% of all knee related problems seen in primary care [5].

Patellofemoral pain is often considered a ‘diagnosis of exclusion’, in the prescence of anterior knee pain with no other diagnosable pathology [1]. As such, the underlying aetiology is largely unknown, although it is often considered a loading related condition [6]. This is because PFP often presents in highly active populations such as runners and military recruits, and has been associated with altered knee joint loading [7]. Despite this, patellofemoral pain also presents in inactive adolescents [3], and the patterns of pain are often diffuse and highly variable [8], and can persist even when adolescents modify or reduce physical activity [9], which undermines the notion that it is primarily driven by load. Additionally, Boudreau et al. noted that a longer symptom duration was associated with a greater distribution or spreading of the area of pain [8]. Together, these observation have led recent studies to investigate the role of other potential contributors to the pain experience, including psychological factors [10] and pain sensitivity [11]. Studies of pain sensitivity have demonstrated local mechanical hyperalgesia around the patella in adolescent [12] and adult females with PFP [13], while widespread mechanical hyperalgesia has only been reported in young female adults [13,14].

Other adaptive characteristics of the pain system such as pro-nociceptive (e.g. temporal summation of pain) and anti-nociceptive (e.g. conditioning pain modulation) mechanisms have also been assessed in individuals with PFP [11]. Conditioned pain modulation (CPM) is an anti-nociceptive mechanism, which is representative of descending control of pain in the central nervous system. It is thought to occur through diffuse noxious inhibitory control (DNIC) which modulates pain processing at the spinal cord level [15], and used proxy of the effectiveness of the endogenous analgesic system and is evident in long-standing pain conditions such as knee and hip osteoarthritis [16–18], where it is associated with development of post-operative pain, in addition to other non-arthritic chronic pain conditions [19,20]. Interestingly, one study previously identified impared conditioned pain modulation in young females with PFP, but no differences in pro-nociceptive mechanisms (temporal summation) [11]. Little is known about CPM in young adults with PFP.

The primary aims of this study were to compare (i) CPM, and (ii) wide-spread hyperalgesia in young adults with PFP to an age and sex matched pain-free control group. We hypothesised that young adults with PFP would have an impaired CPM compared to pain-free controls, and that PPTs around the patella and at sites remote from the area of self-reported knee pain would be lower among young adults with PFP.

## Methods

This cross-sectional study compared PPT and CPM in young adults diagnosed with PFP to a sex and age-matched comparison group of healthy pain-free controls. The study was approved by the local ethics committee at the University of Queensland, Brisbane, Australia and all participants provided written informed consent. The reporting of the study complies with the ‘Strengthening the Reporting of Observational studies in Epidemiology’ (STROBE) statement [21].

### Recruitment

Participants with PFP were recruited from the University of Queensland campus through advertisements in University News, flyers and verbal information to lecture attendees and from newspaper advertisements in the greater Brisbane area. Participants underwent a short telephone screening to determine eligibility. If participants had anterior knee pain with an insidious onset of more than six weeks they were invited to attend a clinical examination by an experienced, registered physiotherapist. We used similar inclusion and exclusion criteria as Collins et al [22] which aligns with current recommendations [1]. During the clinical examination, individuals were diagnosed with PFP if they met the following criteria: insidious onset and a duration of anterior knee or retro-patellar pain for at least the past six weeks; pain provoked by at least two of the following knee loading activities: squatting, running, hopping, or stair walking and ability to read/understand English. Exclusion criteria were concomitant injury or pain from other body sites, or other knee structures; previous knee surgery; patellofemoral instability; knee joint effusion; use of physiotherapy for knee pain within the previous six months; currently undergoing medical treatment for their knee pain; known neurological or medical conditions.

Healthy pain-free controls who were matched for age and sex were recruited from the same population setting. In addition, participants diagnosed with PFP were asked to invite a friend with no knee pain to participate. The inclusion criteria for pain-free controls were: no current self-reported musculoskeletal pain; no self-reported prior surgery in the lower extremity; no self-reported neurological or other medical conditions.

### Outcomes

The *a-priori* defined primary outcome was the percentage change in mechanical PPT from before application of a conditioning stimulus to immediately after. Secondary outcomes included the baseline assessment of mechanical PPT at the three test sites before the conditioning stimulus.

All mechanical PPT measurements were performed by a female investigator blinded to whether participants were diagnosed with PFP or were pain-free controls. The measurements were collected from the most painful knee and shin and contra-lateral elbow. All participants were asked to refrain from strenuous exercise and avoid taking pain medication for 48 hours prior to assessment. Mechanical PPTs were measured with a hand-held pressure algometer (Algometer Type II; Somedic AB, Sweden). Identical to a previous study [23], we measured mechanical PPTs at one site on the centre of the patella, one site on the tibialis anterior (defined as the muscle belly of the tibialis anterior 5 cm distal to the tibial tuberosity) and a control (remote) point on the lateral epicondyle of the humerus. The probe of the mechanical algometer was 1cm^2^, and placed perpendicular to the skin during the PPT measurements. Pressure was applied at a rate of 30 kPa/s and participants were instructed to indicate when the sensation changed from a sensation of pressure to the first onset of pain. Measurements were done with the participants resting in a reclining position and the knee flexed to approximately 60 degrees. Mechanical PPTs was measured twice at each site and the average was used for further analysis.

For the assessment of CPM, a painful conditioning stimulus was applied to the hand, contralateral to the most painful knee, by immersion into a cold water bath. The test stimulus consisted of PPTs tested prior to, and immediately after the conditioning stimulus. The CPM was defined as the percentage change in PPTs (test stimulus) from before to immediately after the conditioning stimulation. The conditioning stimulation was induced by immersion of the hand into a cold water bath (12 degrees, non circulating) for two minutes. Based on pilot testing, we expected this conditioning stimulus to cause the participant to report at least 4–6 out of 10 on a Numeric Rating Scale (NRS) of pain. Just before removing the hand from the water, the participant was asked to rate their pain intensity on a 0–10 NRS, with 0 defined as “no pain” and 10 as “worst pain imaginable”.

### Self-reported pain and disability

In addition to PPTs, the following clinical self-reported measures were collected from the group with PFP by the person doing the physical assessment of eligibility: 1) Knee Injury and Osteoarthritis Outcome Score (KOOS) with scores ranging from 0 (worst) to 100 (best) and covers the five domains: pain, symptoms, function in daily living, function in sport and recreation, and knee-related quality of life; 2) Kujala is a frequently used validated outcome measure for patients with PFF [24] where a maximum score of 100 represents fully functional and lower scores indicate greater pain and/or disability; 3) Numerical Rating Scale (NRS) for worst pain intensity during the last week; 4) symptom duration (months); 5) most painful knee (right/left); 6) uni- or bilateral pain (yes/no) and the pain localisation.

### Pain localisation

A modified version of The Knee Pain Map (KPM) was used to describe pain location and pain distribution [25]. The KPM is an interviewer-administered survey that instructs patients to point to the area of pain. The participant sat on an examination table with knees flexed over the edge of the table, and pointed at or covered the area or areas that hurt with the fingers or hand. The interviewer recorded the areas of pain on the knee diagram (map). Following the survey, the same assessor interpreted all the drawings. Based on the drawings, pain localisation was classified as retropatellar (on the patella), peripatellar, or a combination of both retropatellar and peripatellar. In addition, if the pain extended over a region two fingers width in size or smaller, it was defined as localised knee pain. If the pain covered an area more than two but less than four fingers width in size, it was defined as regional knee pain. If the participant felt the pain was spread over an area larger than this or said that they felt the pain all around and on the patella, the pain was defined as diffuse knee pain. If participants with PFP reported more than four local areas of pain or more than two regions of pain in the knee, the pain was classified as diffuse.

### Reliability

Before collecting data for the study, seven participants (two with PFP and five healthy) participated in an intra-day and intra-tester test-retest reliability study. There was one hour between each test. The reliability of PPT measures for each of the three sites were >0.98 (ICC 3.1, two-way mixed agreement model) and the reliability of the change in PPT after the conditioning stimulation was >0.85 (ICC 3.1, two-way mixed agreement model) for all three sites.

### Sample size

The sample size was calculated based on a difference between groups of 50 kPa in a change on the primary outcome of PPT around the knee after the conditioning stimulus (corresponding to a 50 kPa change in pain-free and 0 kPa change among individuals with PFP). The common standard deviation was estimated to be 75 kPa. Using a power of 0.80 and alpha at 0.05 this corresponds to a sample size of 35 in each group.

### Statistical analysis

The primary analysis was a comparison between groups in the mean percentage change in PPT at the three test sites after the conditioning stimulation was applied. Secondary analysis included a comparison of PPT at each of the three sites between groups.

A two-way ANOVA was used with group (PFP, controls) and test site (patella, tibialis anterior, lateral epicondyle) as factors to test the difference between groups in CPM and PPTs. This analysis was also conducted as an ANCOVA with sex as a covariate. All calculations were performed using Stata version 11 (StataCorp, College Station, Texas, USA). Mean values ±SD are reported if data are normally distributed. If data are non-normally distributed they are presented as median and interquartile range (IQR).

## Results

### Participants

The adults with PFP (n = 33) had a median symptom duration of 24 months (Table 1). The knee pain map showed that most participants with PFP reported retropatellar pain (n = 18) or combined peripatellar and retropatellar pain (n = 12), with only one reporting peripatellar pain. Thirty adults with PFP participated at least weekly in recreational sporting activities above 30 minutes with 22 reporting a decrease in sports participation after they developed knee pain. The KOOS subscale scores were: KOOS-pain (75±12), KOOS-symptoms (83±10), function in daily living (86±11), function in sport and recreation (60±20) and knee-related quality of life (QoL) (51±20) and the Kujala score was 76±8.

**Table 1:** Demographics and patient reported outcomes (presented as mean and standard deviation).

	Patellofemoral pain (n = 33)	Pain-free controls (n = 32)	P-values
**Sex (F/M)**	23/10	22/10	0.93
**Age [years]**	28.5 (5.3)	27.1 (5.2)	0.29
**Weight [kg]**	69.7 (16.3)	63.9 (13.1)	0.12
**Height [m]**	1.69 (0.11)	1.70 (0.09)	0.63
**Body Mass Index (BMI)**	24.2 (3.6)	21.9 (3.0)	0.001
Duration of symptoms (months)*	24 (14–60)	N/A	N/A
Worst pain last week [NRS]*(0–10)	5 (3–7)	N/A	N/A
**Most painful knee (left/right)**	21/12	N/A	N/A

* Median and interquartile range

### Between-group comparison

**Fig 1:**
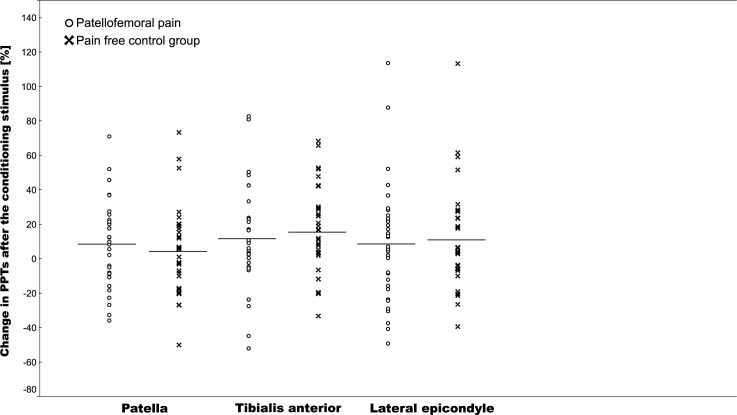
Percentage change in PPTs after the conditioning stimulus across the three test sites (individual patient data and group mean).

**Fig 2:**
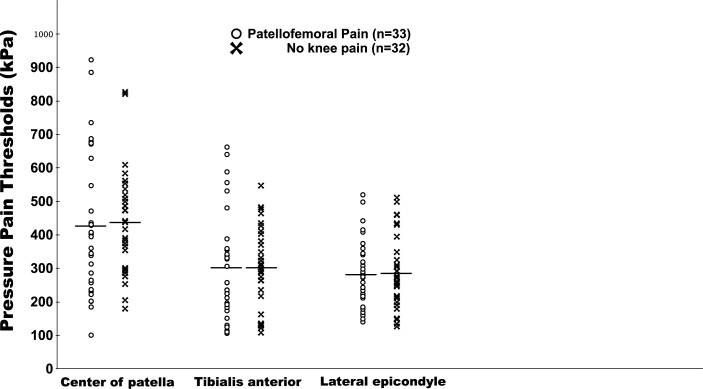
Mechanical PPTs at the three test sites before the conditioning stimulus.

The CPM testing induced a significant increase in PTTs across the three sites (mean CPM range: 6.3–13.5%, P<0.05), but there was no difference in CPM between the PFP and control groups, (*F*(1,189) = 0.39, P = 0.89, Fig 1, mean values presented in S1 File) nor when adjusting for sex using an ANCOVA. There was no difference in mechanical PPTs between the two groups (*F*(1,189) = 0.03, P = 0.86), Fig 2, mean values presented in S1 File). There was a mean increase in pain NRS of 7 (IQR: 6–8) after the hand was immersed into cold water (the conditioning stimulus).

### Explorative analyses within the PFP group

An explorative analysis (not pre-defined) of the association between PPT and self-reported measures of KOOS QoL, KOOS pain, worst pain intensity in the previous week, and symptom duration, revealed no significant or relevant associations (spearman rank correlation<0.31, P>0.08).

**Fig 3:**
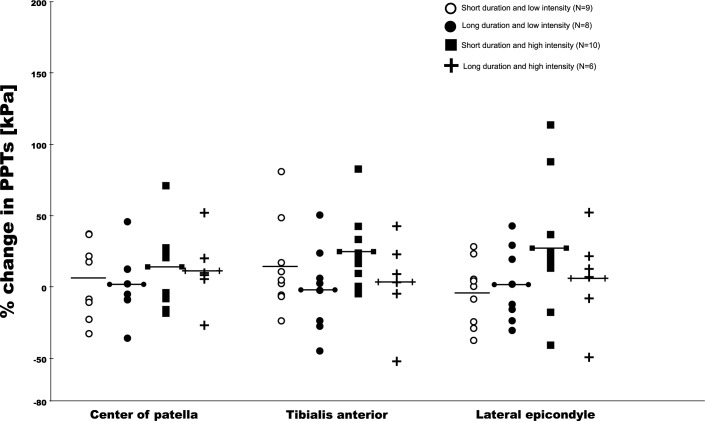
Change in PPTs after the conditioning stimulus at the three test sites split into four groups based on a median split of pain intensity and duration of symptoms among patients with PFP.

**Fig 4:**
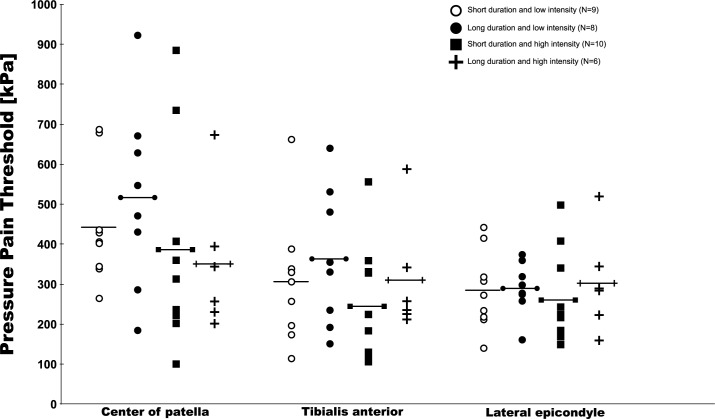
Mechanical PPTs at the three test sites split into four groups based on a median split of pain intensity and duration of symptoms among patients with PFP.

After a median split of symptom duration (at 24 months) into short and long duration, and worst pain intensity (at 5 NRS) into low and high intensity pain, in order to create four distinct subgroups, there were clear patterns towards lower PPTs among the two groups of patients with the highest pain intensity (Figs 3 & 4). As these groups were very small, no formal hypothesis testing was done and this should only be considered explorative to inform future studies.

## Discussion

Contrary to our hypotheses, we found no difference in CPM or PPT at the knee, tibia and elbow between adults with PFP and age and sex matched pain-free controls. Collectively, these results suggest that this group of patients with PFP does not have altered peripheral or central processing of pain compared to age and sex matched pain-free controls.

### Explanation of results

The participants in this study all experienced knee pain during activities that load the patellofemoral joint. They had less severe knee pain, as indicated by lower pain intensity, shorter symptom duration and better KOOS scores, compared to participants in previous studies [11,13,26]. The lower severity might have been reflected in their PPT scores. This is supported by the subgroup analysis stratified on pain intensity and symptom duration, where the lowest PPT scores were seen among patients with the highest pain intensity. This is similar to Coombes et al who showed that higher severity of symptoms in lateral epicondyalgia was linked with larger differences in mechanical hyperalgesia [6]. The explorative plot in Fig 4 demonstrating the percentage change in PPTs after the conditioning stimulus does indicate that those with the shortest duration of symptoms experience the largest changes in PPTs. Collectively, this may lend further support to previous notions of a graded involvement of the nervous system based on symptom severity [27].

Participant age is another key factor differentiating the current study from previous publications. Our participants were older than in prior studies and they developed pain during their mid-twenties compared to previous studies that included participants who developed pain during adolescence. Emerging evidence suggests that adolescence and childhood may be critical periods where pain experiences induce long-lasting effects not observed among adults [28].

Mechanical PPTs measure hyperalgesia of superficial structures and muscles. Measuring hyperalgesia directly from deep chondral bone in patients with PFP could provide new insights into the manifestiations of peripheral and central manifestations of pain. However to our knowledge, such a method does not exist yet.

### Comparison to previous studies

Young female adults (age 20) with long-standing patellofemoral pain (average of 6 years) demonstrated impaired an CPM response compared to pain-free healthy controls [23]. In addition to age and symptom duration, the study by Rathleff et al used a cuff pressure around the lower leg as the conditioning stimulus. The type of conditioning stimulus can influence the CPM effect. Oono et al. [29] recently showed that CPM paradigms with different test stimuli may yield very different results. They found that among the same participants, CPM may range from 0.5% and up to 37%. The strongest CPM effect was observed using a cold pressor test similar to the present study while a lower effect was observed with a cuff pressure. Despite using a potent conditioning stimulus (CPM range: 6.3–13.5%), we did not observe an impaired CPM in this population compared to controls.

### Strengths and limitations

Before we conducted the study, we assessed the reliability of the PPT outcomes and CPM paradigm. Our methods proved to be reliable which suggest that lack of differences in outcome measures between groups was not just attributed to error. Likewise, we used a blinded outcome assessor to minimize the risk of detection bias. We did not collect a blinding index for the outcome assessor, which makes it difficult to ascertain the success of blinding. Due to time constraints, we recruited 5 subjects less than planned 70. This is unlikely to affect any of our conclusions.

We recruited participants who volunteered through advertising. These participants were not recruited through their contact with a medical practitioner or physiotherapist. We did not ascertain if they were actively seeking treatment, which might limit generalisation of this data to those seeking intervention. While the pain levels are lower than other studies, arguably a mean 5/10 pain NRS (IQR: 3 to 7) in our PFP participants is of the order expected to be of concern to the individual and underpins the relevance of our findings. To further investigate the association between symptom severity and pain sensitisation, larger sample-sizes are needed. As this study was not designed as an equivalence study, larger studies are needed to confirm the lack of difference between groups within a population similar to the current.

## Conclusion

We found no difference in CPM between adults with PFP and age and sex matched pain-free controls. Collectively, these results suggest that this group of patients did not exhibit alterations in peripheral or central processing of pain.

## Supporting information

S1 File Mean values for pressure pain thresholds and conditioned pain modulation. (XLSX)

S2 File Supporting data for results. (XLSX)
